# Simultaneous electrochemical solar power generation and storage using metanil yellow-formic acid as a new sensitizer-reductant couple in photogalvanic cells†

**DOI:** 10.1039/c8ra10014d

**Published:** 2019-03-06

**Authors:** Pooran Koli, Yashodhara Dayma, Ramesh Kumar Pareek

**Affiliations:** Department of Chemistry, Jai Narain Vyas University Jodhpur 342001 Rajasthan India poorankoli@rediffmail.com poorankoli@yahoo.com +91 291 2614162

## Abstract

With the rapid commercialization of solar and wind power as supplements and potential substitutes of fossil fuels, the need for power storage techniques to render renewable energy sources impervious to climatic variations has gained significant importance recently. In addition to this requirement of power storage, photo-galvanic (PG) cells hold special significance because these photo-electrochemical devices are capable of simultaneous solar power generation and storage. PG cells with performance as high as 649.6 μW power (*P*_pp_), 2250 μA current (*i*_sc_), 1048 mV potential (*V*_oc_), 8.12% conversion efficiency (CE), and 59 minutes power storage capacity (as half-time, *t*_0.5_) have been reported under artificial and low illumination intensities. To enable PG cells, a future source of solar energy conversion, with storage as well, their efficiency must be improved further to a level comparable to that of photovoltaic cells. The metanil yellow dye (photo-sensitizer)-formic acid (reductant) couple has not been exploited to date for this purpose. Therefore, in the present study, the metanil yellow dye as a photosensitizer and formic acid as a reductant have been used in the presence of sodium lauryl sulfate surfactant and sodium hydroxide alkaline medium to further increase the solar energy conversion efficiency and storage capacity of PG cells. The present study reports greatly enhanced electrical performance (compared to earlier results for similar cells) of *P*_pp_ 822 μW, *i*_sc_ 6000 μA, *V*_oc_ 1110 mV, CE 20.41%, and *t*_0.5_ 105 minutes. On the basis of the redox potential and reported data, a plausible mechanism has also been proposed for the photo-generation of current in metanil yellow-formic acid photogalvanics.

## Abstract 2

Variation of the pH. (1) Current *vs.* pH (lower curve); (2) power *vs.* pH (upper curve).

## Graphical abstract

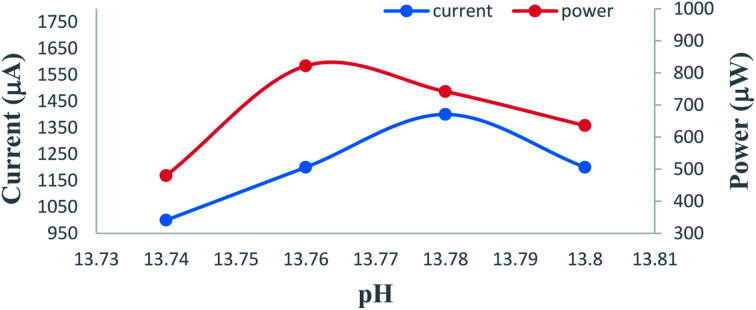

## 1. Introduction

A photogalvanic (PG) cell is a device in which light is absorbed by an electrolyte solution containing a sensitizer, giving rise to a high energy excited sensitizer species. These high energy species lose energy electrochemically, leading to solar power generation and storage. The PG cell is a prominent example of photo-electrochemical systems consisting of anodic and cathodic electrodes dipped in a solution mixture of compulsory chemicals such as photo-sensitizer(s), reductant(s) and an alkali. A surfactant (an optional chemical) may also be added to this solution mixture to enhance the performance of the PG cell by increasing the solubility and stability of the dye sensitizer. PG cells are based on the photo-galvanic effect, in which light has an influence on the electrode potential due to a photochemical process in the bulk of the electrolyte. The photogalvanic effect is a special type of Becquerel effect (in the Becquerel effect, photochemical or photoelectric processes occur on the surface layer of the electrode).^1^ The photogalvanic effect was first detected by the action of light on the equilibrium of ferrous iodine-iodide.^2^ This effect was first systematically studied through the photogalvanic properties of the thionine-iron system by Rabinowitch,^3^ who suggested that this photogalvanic effect could be used for solar energy conversion and storage. To put this suggestion into application, various studies on photo-galvanic cell systems have been reported.^4–33^ The use of various dye photo-sensitizers, such as thionine,^3^ methylene blue,^4^ thionine-loaded Nafion film,^5^ bromophenol red,^6^ fuchsine basic,^7^ Congo red,^8^ azure A,^10^ and malachite green;^11^ various inorganic and organic reductants, such as iron,^3^ ethylenediamine tetraacetate-EDTA,^6–8^ ascorbic acid,^10^ and arabinose;^10^ and various surfactants, such as NaLS,^10,11^ Tween-80,^13^ diethylenetriaminepentaacetic acid (DPTA),^17^ and dioctylsulphosuccinate (DSS)^20^ have been reported for solar energy and conversion through PG cells. A literature survey reveals that the main fabrication components of PG cells are an anodic electrode (Pt), cathodic electrode (saturated calomel electrode-SCE), sensitizer(s), reductant(s), NaOH, H-shaped glass tubes, *etc.*, and the optimum electrical cell performance is dependent on variables such as concentration (of the sensitizer, reductant, surfactant, and NaOH, *i.e.*, pH), diffusion length, electrodes, electrode kinetics, diffusion, external load, illumination intensity, and temperature. Therefore, to further improve the electrical performance of PG cells, these variables must be the centre of study. To date, studies have reported the photo-decay of dye sensitizers to be a hurdle causing low cell efficiency. Therefore, a dye with a stable framework must be used along with a good reductant. The azo-aromatic dye metanil yellow has been reported to be a good photodecay-resistant dye, probably because its aromatic groups have strong aromatic bonds and are difficult to degrade.^34^ It is also a low molecular weight and inexpensive dye with prospects for higher diffusion (a favourable factor for the power output of PG cells) and low-cost cell technology. Further, formic acid has been reported to be an extremely stable, safe organic acid with good reduction capacity of various species due to its combined acid/aldehyde character and the possibility for exothermic release of carbon dioxide on hydride donation.^35,36^ The literature survey also reveals that the metanil sensitizer-formic acid reductant couple has not attracted the attention of photogalvanicists to date.

It is also reported that the use of alkaline medium and small Pt electrodes^19^ with surfactants^25^ enhances the efficiency of PG cells. Anionic surfactants are reported to be the most effective surfactants for solar power and storage through PG cells.^7,26^ In view of these facts, a metanil yellow-formic acid-sodium lauryl sulfate (SLS)-NaOH chemical system along with a small Pt electrode and SCE component of the combination electrode has been used to further enhance the electrical output of PG cells at low intensity. At optimal values of the cell fabrication variables, the *i*–*V* characteristics of the cell show that the highest power (*i.e.* 822 μW) is extractable from the cell, with a conversion efficiency of 20.41% and a power storage capacity of 105 minutes. The results of the present study at low illumination intensity are sufficiently novel to report advancement over previous results, *viz.*, power 649.6 μW, conversion efficiency 8.12%, and power storage capacity 59 minutes.^37^

## 2. Materials and methods

The chemicals metanil yellow (70% assay-purity), formic acid (HCOOH), sodium lauryl sulfate (NaC_12_H_25_SO_4_), and sodium hydroxide (NaOH) were used as the photo-sensitizer, reductant, surfactant, and alkaline medium, respectively. Solutions of metanil yellow dye (M/500), formic acid (M/100), sodium lauryl sulfate (M/10), and sodium hydroxide (1 M) were used. All these solutions were prepared in single distilled water and maintained in amber vessels to protect them from sunlight. The experimental setup consisted of an H-cell (photogalvanic cell) with a model 335 digital pH meter (for measuring the potential in millivolt-mV) manufactured by Systronics India Ltd., Ahmedabad, India; a micro-ammeter (for measuring the current in microamperes, μA) manufactured by the OSAW company, Haryana, India; carbon pot log 470 K devices (for changing the resistance of the circuit), and a circuit key (Fig. S1†); the efficiency and fill factor were calculated using formulas^19,20,38^ [see Sections 1 to 9 of the ESI†].

## 3. Results and discussion

**Fig. 1:**
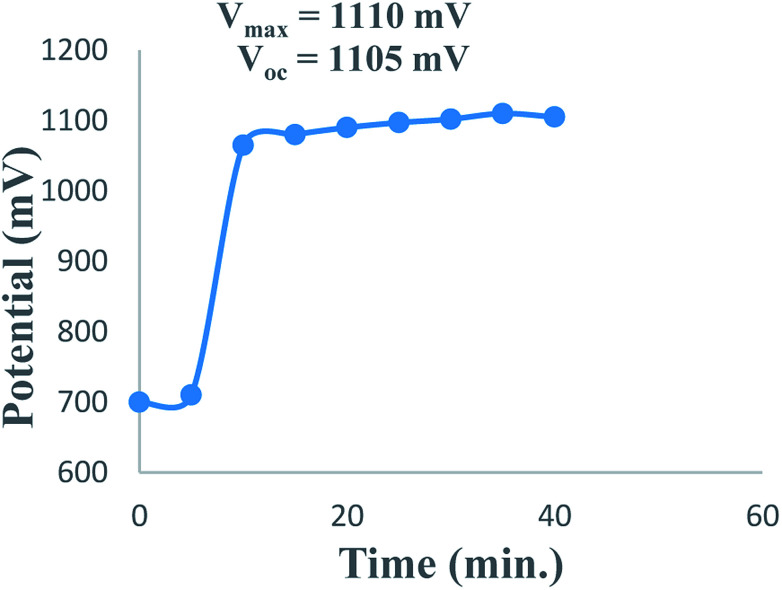
Variation of the potential with time during charging of the PG cell.

The overall performance of 28 PG cells containing metanil yellow dye as the photo-sensitizer, formic acid as the reductant and SLS as a surfactant in alkaline medium was observed. The performance of each PG cell was determined with respect to the electrical output, initial generation of photocurrent, conversion efficiency, storage capacity of the cell system, *etc.* On illuminating each PG cell, the potential increased regularly and reached the highest value (*V*_max_), which then decreased and became quite constant (*V*_oc_) after some time (Fig. 1). This can be attributed to the increase in the number of excited and electron-donating dye molecules during illumination. At *V*_max_, the number of excited and electron-donating dye molecules is the highest. The theoretically highest possible *V*_oc_ for a band gap of 3.0 eV is 2.6 V. In the present case, the observed *V*_oc_ for metanil yellow (band gap ∼2.99 eV, corresponding to *λ*_max_ ∼411 nm at ∼10^−5^ M concentration of metanil yellow) was only 1.1 V. However, it should be noted that for dye-sensitized solar cells such as PG cells, a low *V*_oc_ may be one of the factors contributing to their relatively low efficiency in comparison to that of PV cells. The low *V*_oc_ can be attributed to the non-optimized energy levels of the metanil yellow dye sensitizer with respect to the redox potential of the electrolyte.^39^

**Fig. 2:**
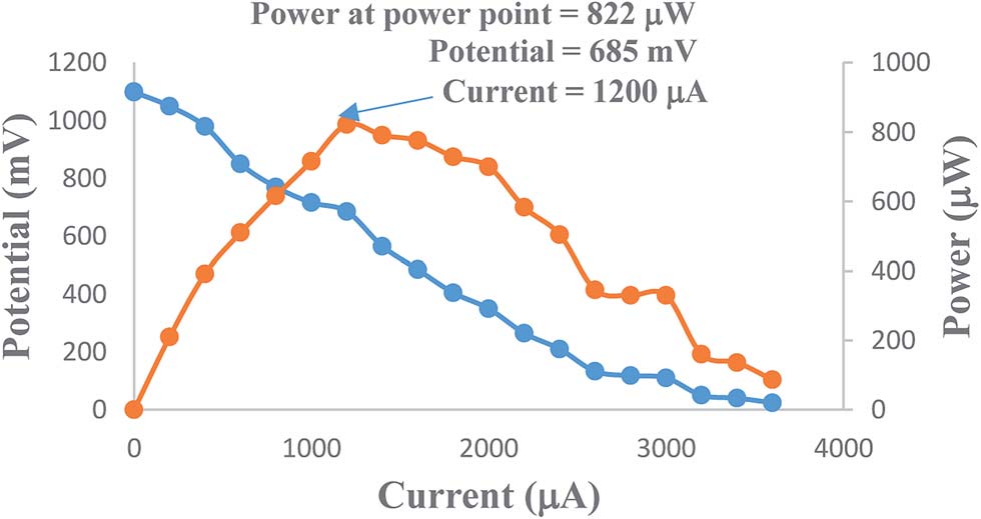
Variation of the potential and power with the *i*–*V* characteristics of the cell. (1) Potential *vs.* current (nearly linear curve). (2) Power *vs.* current (inverted, nearly V-shaped curve).

The *i*–*V* characteristics of the PG cell (Table S1,†Fig. 2) show that the highest power is obtained at a characteristic current. The *i*–*V* characteristics indicate that 822 μW, the highest power at the power point, is obtainable at a current of 1200 μA. The optimum cell performance at optimal values of the cell variables is summarized as dark potential 509 mV; maximum potential (*V*_max_) 1110 mV; open-circuit potential (*V*_oc_) 1105 mV; maximum current (*i*_max_) 6000 μA; short-circuit current (*i*_sc_) 3600 μA; conversion efficiency (CE) 20.41%; and fill factor (FF) 0.206. The study of the effects of the variation of various variables, such as the concentrations of metanil yellow dye, formic acid, SLS, and NaOH (*i.e.*, in terms of pH), shows that the values of these variables affect the electrical performance of the cell at artificial sunlight intensity, and the cell has optimum performance at optimal values of the variables.

### 3.1. Mechanisms of solar power generation and storage

The photo-electrochemistry of the formic acid reductant solution alone, pure SLS surfactant solution alone, and the solution mixture of metanil yellow dye sensitizer with formic acid reductant and NaOH alkali were studied in the PG cell to propose a plausible mechanism in light of previously published literature. The illumination of the solution containing metanil yellow dye sensitizer along with the formic acid reductant and NaOH alkali shows the generation of the photo-potential and photo-current.

The pure formic acid reductant solution showed a potential of −136 mV (*vs.* SCE) and zero current in the dark. The illumination of this formic acid reductant solution showed no change in potential or current, indicating that formic acid is neither an electro-active species at the electrodes (it does not donate/accept electrons to/from Pt/or SCE) nor a charged carrier in the solution. The lack of change in the potential of pure formic acid on illumination (same potential in both dark and light conditions) is justified by the fact that there is no photo-excitation of electrons in formic acid because formic acid does not absorb light with wavelengths above ∼165 nm, *i.e.*, below ∼7.5 eV;^40^ in the present case, an illuminating source of 300 nm to 3000 nm was used.^19^

However, the metanil yellow dye in the presence of the formic acid reductant showed photo-potential and photocurrent, indicating its electro-active nature and the electron exchange between metanil yellow dye molecules and formic acid molecules; this, in turn, shows the formation of the reduced and oxidized states of formic acid. This fact is supported by published literature, where the reductant in its reduced and oxidized forms behaves as an electron carrier and diffuses through paths in the cell.^24^ The metanil yellow dye with formic acid reductant shows photo-potential and current, indicating that the metanil yellow dye is the electro-active species. The scientists Wildes and Lichtin reported studies on the effects of diffusion path length on the current parameters; they concluded that the leuco (two electrons-reduced structure) or semi (one electron-reduced structure) reduced forms of the dye and the dye molecule itself are the main electroactive species at the illuminated and dark electrodes, respectively.^22,23^ On this basis, the leuco-reduced excited states of metanil yellow dye and the metanil yellow dye itself are supposed to be the main electroactive species at the illuminated and dark electrodes, respectively.

The energy stored in the charge-separated leuco metanil yellow form is converted into electrical energy by the so-called photogalvanic effect. The formic acid reductant donates an electron to the leuco metanil yellow sensitizer, and the excess electrons on the sensitizer molecules are transferred to the Pt electrode.^25^ The photoinduced electron transfer from the surfactant to the dye molecule through a charge transfer interaction enhances the process of electron transfer to the photosensitizer^26^ and, in turn, to the Pt electrode, leading to higher current and cell performance.

Some electrochemical studies on metanil yellow dye have reported that reduction of the azo group is a two-step diffusion-controlled irreversible process involving two electrons;^41^ this is suggestive of an irreversible process based on the diffusion-controlled leuco electroactive species of metanil yellow dye in the present photogalvanics study.

**Scheme 1:**
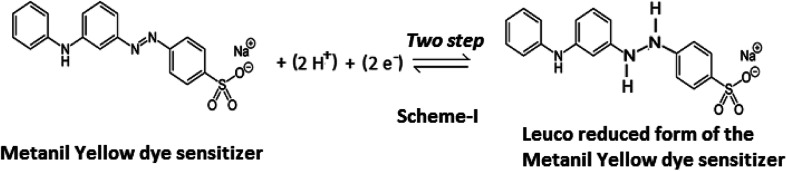


In the present study of the azo dye metanil yellow, the metanil yellow dye molecule itself and the two electrons-reduced leuco form of the metanil yellow dye molecule should be the electro-active species at the SCE and Pt electrodes, respectively (Scheme 1).

The fact that the electrode process involves two-electron and two-proton reduction of the azo group (–N=N–) of the metanil yellow dye molecule to the hydrazo compound is supported by a reported cyclic-voltammetric study in aqueous solution. It is reported that reduction of the azo group of metanil yellow is a two-step process at an average cathode reduction potential of −0.328 V (Pt *vs.* SCE electrode; *i.e.*, −0.293 V at a scan rate of 50 mV s^−1^ and −0.363 V at a scan rate of 2000 mV s^−1^) with a corresponding average Pt anode oxidation potential of −0.72 V (Pt *vs.* SCE electrode; *i.e.*, −0.697 V at a scan rate of 50 mV s^−1^ and −0.743 V at a scan rate of 2000 mV s^−1^). A cyclic-voltammetric study has reported that the electrochemical reactivity of metanil yellow dye is dependent on pH and is a diffusion-controlled, irreversible process, as indicated by the linear increase in the cathode peak current with half potential.^41^

It is also clear from this reported study that the average reduction and oxidation potentials of metanil yellow dye at Pt electrode (*vs.* SHE) are ∼+0.088 V and ∼0.961 mV, respectively. From the redox reaction (Scheme 1), it is also clear that the redox potential of metanil yellow is pH dependent; also, the 0.092 V reduction potential and 0.929 V oxidation potential (*vs.* SHE) of metanil yellow dye in the ground state and excited state, respectively, observed in this photogalvanic study in aqueous medium are supported by cyclic voltammetry studies in the literature.

In a PG cell, the photon energy is not lost and degraded to heat but is involved in endergonic processes, occurring as the storing of quantum energy in the form of labile intermediates, *i.e.*, semi/leuco transition states.^42^ Higher delocalization of electrons and bulkiness of the dye will enhance the stability of the excited states of the dye, which in turn will lead to a higher storage capacity. Recombination processes may lead to a decrease of the storage capacity.^43^

The solution of the anionic surfactant SLS alone shows a potential of −281 mV (*vs.* SCE) and a current of ∼10 μA in both the dark and illuminated states. This shows that the current and potential of SLS are due to its anionic charge diffusion in the cell and its somewhat electro-active nature. The illumination has almost no effect on the potential of SLS because SLS has no ability to absorb the photons emitted at 300 nm to 3000 nm from the 200 wattage bulb used in the present study. This fact is supported by the published literature, which indicates that SLS has no absorbance above 210 nm (below this energy) and that the absorbance below 210 nm is also negligible in the concentration range of 14 mM ^44^ used in the present study.

Further, the potentials (*vs.* SCE) of metanil yellow in the presence of reductant and SLS in dark and illuminated conditions are −509 mV (*i.e.*, *V*_dark_) and −1105 mV (*i.e.*, *V*_oc_), respectively. By adjusting for the potential of the reductant and surfactant, the ground state and excited potentials (*vs.* SCE) of metanil yellow dye are found be −92 mV and −688 mV, respectively. With this calculation, the redox potentials (*vs.* standard hydrogen electrode, SHE) of SCE, ground state metanil yellow dye, formic acid reductant, excited state metanil yellow, and Pt^2+^/Pt are +0.2415 V, +0.3135 V, +0.377 V, +0.929 V, and +1.18 V, respectively. We know that a higher redox potential results in a higher tendency of electrons to exist in the reduced state. This means the electrons will flow from SCE towards Pt in this series in the bulk of the electrolyte. The band gap for metanil yellow is on the order of 2.99 eV, equivalent to *λ*_max_ 414 nm, required for electron excitation from its highest occupied molecular orbital (HOMO) to its lowest unoccupied molecular orbital (LUMO).^45^ In an illuminated region of the cell, a photon excites an electron from a ground state orbital (HOMO) to a higher energy orbital (LUMO) (*i.e.*, the excited singlet state, which collapses to the excited triplet state through inter-system crossing (ISC)) of metanil yellow dye photosensitizer. The formation of the excited state leaves a vacancy in the ground state that can be filled by an electron donor such as formic acid reductant because the redox potential of the excited metanil yellow dye state is higher than that of the formic acid reductant. The net result is that an excess electron is produced in the higher energy state of the metanil yellow dye molecule. The excited state metanil yellow dye molecule cannot hold this excess electron for long; therefore, this electron can be donated to an electron exchanger (Pt electrode). The electrons from the Pt electrode (high potential) flow through the external circuit to SCE (low potential), resulting in the conversion of light into electricity.^19,46^ At SCE, the metanil yellow dye molecules in solution accept electrons because the redox potential of the ground state metanil yellow dye is higher than that of SCE. In this way, the photogalvanic cell enables solar energy conversion into solar power (dc current) with inherent storage capacity. The current carrier in the electrolyte is the diffusion-controlled ions. Ideally, the photogalvanic system acts as a (cyclic) light-driven electricity generator.^24^ The photogalvanic behaviour has been found to be reversible for several cycles.^47^ This reversible behaviour is subject to the limitations imposed by the second law of thermodynamics. The ideal reversibility can be achieved only when the loss of power is zero, and that can occur only when the resistance, friction, dissipation, *etc.*, are zero; however, this position is impossible. Therefore, the element of irreversibility in photogalvanics is intrinsic. This is also evident from a study performed by the scientists Vos and Powels; they stated that open-circuit operation is a reversible process like a Carnot engine and has zero efficiency, but during operation at maximum power at the power point to obtain maximum efficiency, the process is not reversible.^48^ Hillson and Rideal also reported that the quantum efficiency of the photoreaction is independent of the electrical resistance of the circuit only at a small photo-potential.^49^ Murthy and Reddy have also widely studied the photogalvanic activity of various dyes with both reversible and irreversible reducing agents; they reported that cyclic voltammograms show that dye reduction is reversible at low pH and quasi-reversible at high pH, where the protonation chemical reaction may be limiting the charge-transfer process.^50^

The reversibility of the reductant was reported in the metallic redox couple Fe^2+^/Fe only (especially in a thionine-iron system) in initial studies.^1,51^ Later studies used organic reductants such as EDTA, oxalic acid, and mannitol; Hendrich used ascorbic acid as an irreversible reductant.^52^ This means that the organic reductants may undergo photodecay, leading to their sacrifice and, in turn, to their sacrificial and irreversible use. In the present study, formic acid (an organic compound) has been used as the reductant; therefore, its use should be sacrificial in nature.

In the present study, an incandescent bulb (lamp) was used as an illuminating source to charge the photogalvanic cell. The radiation energy of the lamp (tungsten bulb) and about 99 percent of solar radiation energy is emitted in the form of near UV, visible and near infrared radiation (wavelength region from 300 nm to 3000 nm). Metanil yellow dye has a maximum absorbance at *λ*_max_ 414 nm in aqueous medium. Thus, there is overlap between the absorption spectrum of the dye and the emission spectrum of the lamp.

**Fig. 3:**
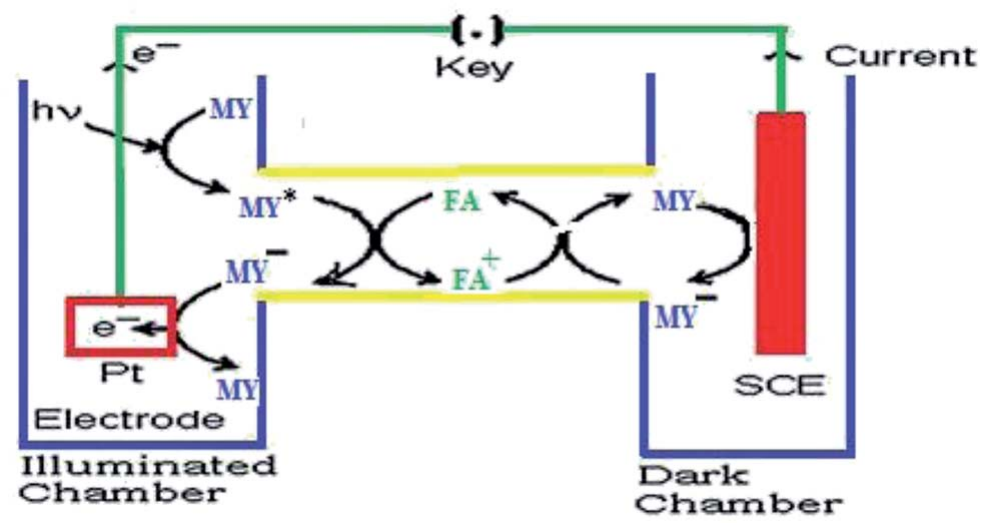
Mechanism of the photo-generation of current in a photogalvanic cell. SCE – saturated calomel electrode; MY – metanil yellow dye sensitizer; MY* – oxidized form of metanil yellow dye; FA^+^ – an oxidized form of the formic acid reductant; e^−^ – electron.

**Fig. 4:**
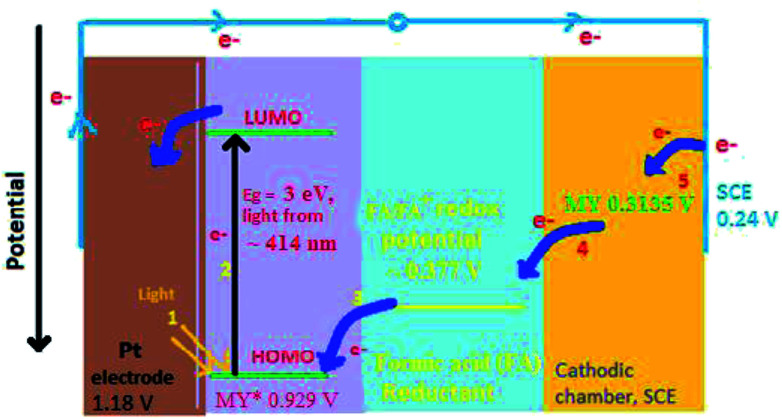
Energy level diagram of photo-generation of current in the cell. MY – metanil yellow dye; SCE – saturated calomel electrode; HOMO – highest occupied molecular orbital of the dye; LUMO – lowest unoccupied molecular orbital of the dye; MY* – excited dye. (1) Absorption of the light at ∼414 nm. (2) Photo-excitation of an electron from the HOMO to the LUMO. (3) Reduction of the excited state dye by electron transfer to the HOMO from the reductant. (4) Transfer of the electron from the reduced ground state dye to the oxidized state of the reductant. (5) Transfer of the electron to the ground state dye from the external circuit through SCE.

**Scheme 2:**
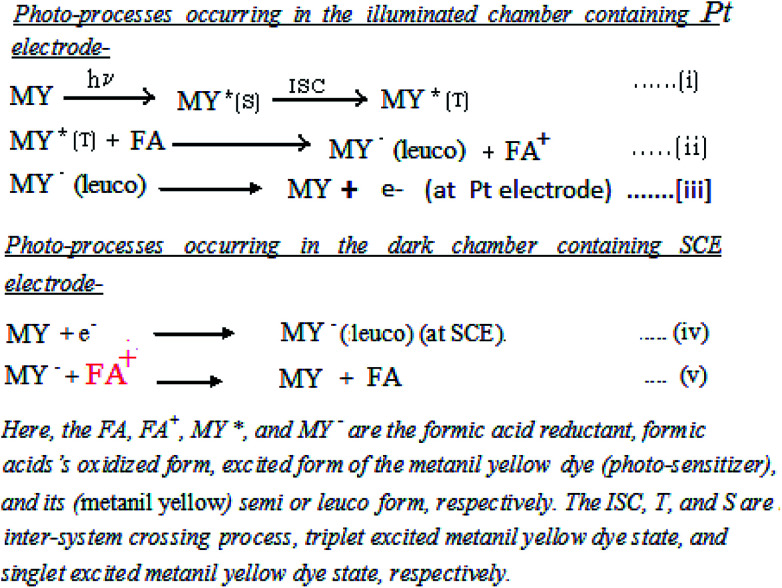


On the basis of the aforementioned facts, the mechanism of photocurrent generation (Fig. 3 and 4) in the photogalvanic cell can be schematically represented as in Scheme 2.^23,51,53^

The *i*_sc_ is the maximum current density achievable upon the generation of only one electron–hole pair from one photon.^39^ The mechanism of photocurrent generation in a photogalvanic cell can be explained as follows:

It is correct that the term “band gap,” which originates from solid-state physics, does not seem to be appropriate for molecular species. However, it should be noted that for materials in inorganic semiconductors (Si, Ge, *etc.*), the band gap is the energy gap between the valence band (*i.e.*, the highest energy band that is occupied by electrons) and the conduction band (*i.e.*, the lowest energy band that is unoccupied by electrons). In the same pattern, organic molecules, such as dye molecules, that are called organic semiconductors have an energy gap between the highest occupied molecular orbital (HOMO, which is equivalent to the valence band) and the lowest unoccupied molecular orbital (LUMO, which is equivalent to the conduction band). Therefore, the energy difference between the HOMO and LUMO of an organic molecule can be treated as a band gap.^39,45^ Thus, 2.99 eV is the real energy of the absorption peak of metanil yellow, which corresponds to the 414 nm wavelength [energy calculated as *E* = *h*(*c*/*λ*)]. The theoretically highest possible *V*_oc_ for a band gap of 3.0 eV is 2.6 V. In the present case, the observed *V*_oc_ for metanil yellow (band gap ∼2.99 eV, corresponding to *λ*_max_ ∼411 nm at a ∼10^−5^ M concentration of metanil yellow) is only 1.1 V.

### 3.2. Effects of variation of the metanil yellow dye photo-sensitizer

**Fig. 5:**
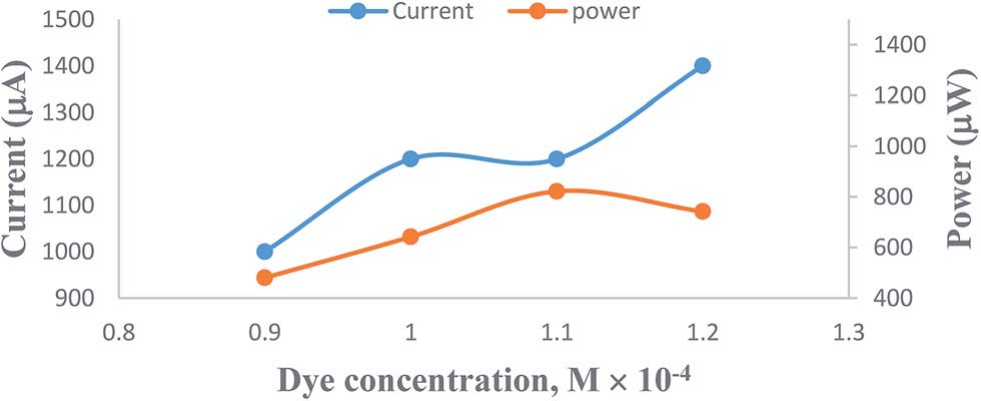
Variation of the photocurrent and power with metanil yellow dye concentration. (1) Current *vs.* concentration (upper curve); (2) power *vs.* concentration (lower curve).

On increasing the concentration of the metanil yellow dye photo-sensitizer, there is an increase in the photocurrent, and the optimum cell performance is observed at 1.1 × 10^−4^ M concentration of the metanil yellow dye (Table 1, Fig. 5, Sec. 10 of the ESI†). This pattern of change in the photocurrent with the metanil yellow dye photo-sensitizer can be attributed to the fact that at a lower concentration range of the metanil yellow dye photo-sensitizer, there will be limited numbers of photo-sensitizer molecules to absorb photons and donate electrons to the Pt electrode in the cell. A higher concentration of the metanil yellow dye photo-sensitizer will not permit the desired light intensity to reach the dye photo-sensitizer molecules near the electrode; hence, there will be a corresponding decrease in the power of the cell. The suitability of the 10^−4^ M metanil dye concentration range for the current generation is supported by a cyclic voltammetric study.^41^

**Table 1:** Effects of variation of the metanil yellow dye photo-sensitizer concentration^a^

Electrical parameters of the PG cell	[Metanil yellow dye] × 10^−4^ M			
	0.9	1.0	1.1	1.2
*i*_max_ (μA)	3200	4000	6000	6000
*i*_sc_ (μA)	2000	2600	3600	3200
Potential at power point (mV)	480	535	685	530
Photocurrent at power point (μA)	1000	1200	1200	1400
*P*_pp_ (μW)	480	642	822	742
Cell charging time (min)	25	25	40	20
CE (%)	12.46	16.97	20.41	18.46
FF	0.216	0.186	0.206	0.207

a At [formic acid] = 1.4 × 10^−3^ M, [SLS] = 1.4 × 10^−2^ M, pH = 13.76, Pt electrode area = 0.4 cm × 0.2 cm, light intensity = 10.4 mW cm^−2^, diffusion length = 5.5 cm.

### 3.3. Effects of variation in the concentration of formic acid reductant

**Fig. 6:**
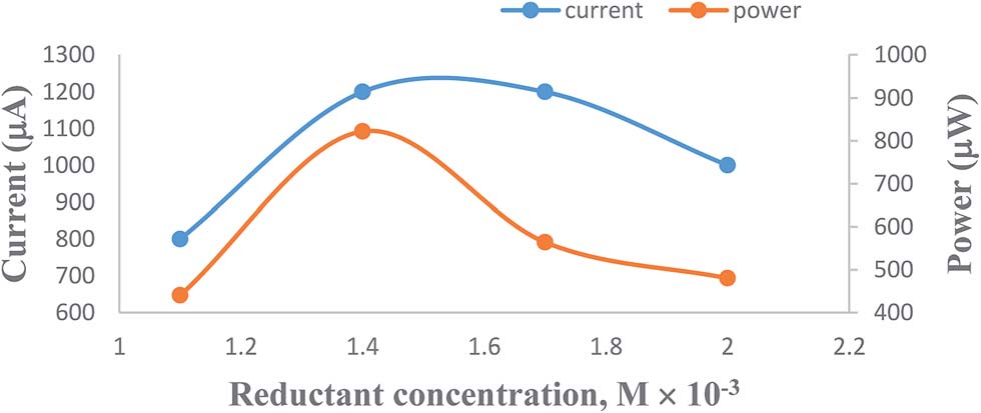
Variation of the formic acid reductant concentration. (1) Current *vs.* concentration (upper curve); (2) power *vs.* concentration (lower curve).

On increasing the concentration of the formic acid reductant at constant values of the other cell fabrication variables, the electrical output of the PG cell was found to increase to a maximum and optimum value; thereafter, it was found to decrease. The optimum cell performance was observed at 1.4 × 10^−3^ M concentration of the formic acid reductant (Table 2, Fig. 6, Sec. 11 of the ESI†). The electrical output is low at a lower concentration range of the formic acid reductant because there are fewer molecules to donate electrons to the excited molecules of the metanil yellow dye photo-sensitizer. A higher concentration of the formic acid reductant may hinder the movement of the metanil yellow dye molecules towards the electrodes in the desired time limit and may also promote back electron transfer from the metanil yellow dye molecules to the formic acid reductant molecules.

**Table 2:** Effects of variation of the formic acid reductant concentration^a^

Electrical parameters of the PG cell	[Formic acid] × 10^−4^ M			
	1.1	1.4	1.7	2.0
*i*_max_ (μA)	3400	6000	3600	3200
*i*_sc_ (μA)	2200	3600	2200	2000
Potential at power point (mV)	550	685	470	480
Photocurrent at power point (μA)	800	1200	1200	1000
*P*_pp_ (μW)	440	822	564	480
Cell charging time (min)	25	40	30	25
CE (%)	10.15	20.41	16.06	12.46
FF	0.192	0.206	0.237	0.216

a At [dye photo-sensitizer] = 1.1 × 10^−4^ M, [SLS] = 1.4 × 10^−2^ M, Pt electrode area = 0.4 cm × 0.2 cm, pH = 13.76, light intensity = 10.4 mW cm^−2^, diffusion length (*D*_L_) = 5.5 cm.

### 3.4. Effects of variation of the SLS surfactant concentration

**Fig. 7:**
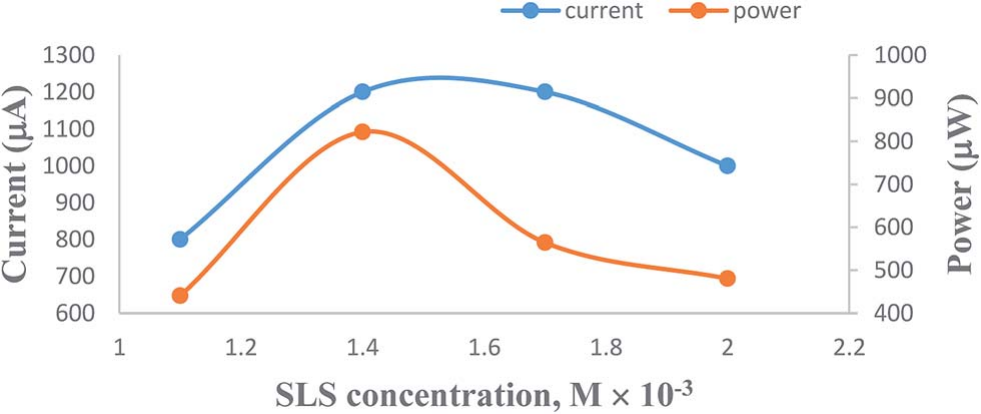
Variation of the SLS surfactant concentration. (1) Current *vs.* concentration (upper curve); (2) power *vs.* concentration (lower curve).

On increasing the concentration of SLS at constant values of all other cell fabrication variables, the electrical output of the PG cell was found to increase to reach a maximum and optimum value. The optimum cell performance was observed at 1.4 × 10^−2^ M concentration of the SLS surfactant (Table 3, Fig. 7, Sec. 12 of the ESI†). At SLS surfactant concentrations lower than 1.4 × 10^−2^ M, the lower value of the electrical output can be attributed to the lower number of SLS surfactant molecules available for electron transfer and the solubility of the metanil yellow dye photo-sensitizer molecules. A higher concentration of the SLS surfactant may hinder the motion of metanil yellow dye photo-sensitizer molecules toward the electrodes, leading to a corresponding decrease in the power of the PG cell. This trend of the effects of SLS on the PG cell can be explained on the basis of certain reported and observed facts. It is a reported fact that SLS and other surfactants have solubilizing effects on dyes. It is a well-known fact that the surfactant solubilizes the dye over its critical micelle concentration (CMC), and this dye solubility increases linearly with increasing surfactant concentration. However, this holds true for moderate concentrations of most surfactants. This is because at very high surfactant concentrations, rod-like micelles are formed, leading to an increase in viscosity. The surfactant-like SLS with a straight alkyl tail provides better solubilization. It is also a reported fact that once micelles have grown to a certain size, the solubilization of dyes is not further facilitated by the micelles becoming even more extended, often ultimately reaching worm-like or thread-like structures.^54^

**Table 3:** Effects of variation of the SLS surfactant concentration^a^

Electrical parameters of the PG cell	[SLS] × 10^−4^ M			
	0.8	1.1	1.4	1.7
*i*_max_ (μA)	4000	4000	6000	3800
*i*_sc_ (μA)	2200	2400	3600	2400
Potential at power point (mV)	460	506	685	525
Photocurrent at power point (μA)	1200	1200	1200	1000
*P*_pp_ (μW)	552	607.2	822	525
Cell charging time (min)	25	25	40	30
CE (%)	14.72	16.27	20.41	12.11
FF	0.222	0.223	0.206	0.192

a At [formic acid] = 1.4 × 10^−3^ M, [metanil yellow dye] = 1.1 × 10^−4^ M, Pt electrode area = 0.4 cm × 0.2 cm, pH = 13.76, light intensity = 10.4 mW cm^−2^, diffusion length (*D*_L_) = 5.5 cm.

The cell power increases with increasing SLS concentration as the dye solubilization increases almost linearly with increasing surfactant concentration beyond the CMC. At very high SLS concentrations, the higher viscosity of the electrolyte can be attributed to the reasons behind the decrease of the diffusion of ions, leading to a decrease in the cell performance. Thus, the reason for these observed effects of SLS concentration on the PG cell can be summarized as follows: on the lower side of the concentration range of SLS, there will be a limited number of SLS molecules to facilitate electron transfer and solubility and stability of the dye; therefore, there is low electrical output at lower concentrations of SLS. Meanwhile, a higher concentration of SLS will hinder the motion of dye molecules towards the electrodes; hence, there will be a corresponding decrease in the power of the cell.

The SLS solution alone also shows a potential value ∼(−) 281 mV and a current ∼10 μA in the dark as well as in the illuminated state. This nature of the contribution of SLS to the current may also be a reason for the enhancement of the cell power in the presence of SLS.

### 3.5. Effects of variation of the alkalinity of the electrolyte medium (*i.e.*, pH)

**Fig. 8:**
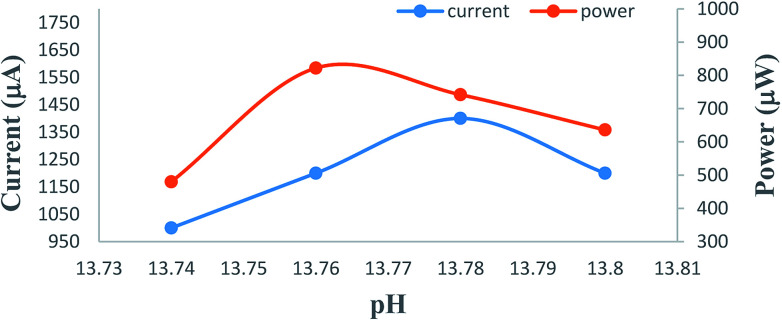
Variation of the pH. (1) Current *vs.* pH (lower curve); (2) power *vs.* pH (upper curve).

It is observed that the PG cell system functions effectively in strong alkaline medium. The photocurrent of the cell is high in the higher pH range. The maximum photocurrent and power at the power point were observed at an optimum pH of 13.76 (Table 4, Fig. 8, Sec. 13 of the ESI†). On further increasing the pH, there is a decrease in the photocurrent; this may be due to the fact that at a very high concentration of OH^−^ (*i.e.*, pH), the OH^−^ may combine with the oxidized state of the formic acid reductant, inhibiting regeneration of its original state. To understand the effects of pH on the cell performance, the possible chemical structures (Scheme 1) and the solubility of the anionic azo dye metanil yellow at low and high pH with certain other reported facts must be taken into consideration. Metanil yellow is an acidic (sulfonic group) dye; thus, it is expected to be in the anionic form even at low pH. Despite this, at very low pH, the availability of the metanil yellow dye molecules in anionic form in the solution will be lowered by the protonation of the sulfonic group and nitrogen atoms, leading to a positively charged dye species with decreased electron donating tendency.

**Table 4:** Effects of variation of the alkalinity (*i.e.*, pH)^a^

Electrical parameters of the PG cell	pH			
	13.74	13.76	13.78	13.80
*i*_max_ (μA)	3200	6000	6000	4200
*i*_sc_ (μA)	2000	3600	3200	2600
Potential at power point (mV)	480	685	530	530
Photocurrent at power point (μA)	1000	1200	1400	1200
*P*_pp_ (μW)	480	822	742	636
Cell charging time (min)	25	40	20	25
CE (%)	12.46	20.41	18.46	16.05
FF	0.216	0.206	0.207	0.210

a At [formic acid reductant] = 1.4 × 10^−3^ M, SLS = 1.4 × 10^−2^ M, [metanil yellow dye] = 1.1 × 10^−4^ M, Pt electrode area = 0.4 cm × 0.2 cm, light intensity = 10.4 mW cm^−2^, diffusion length (*D*_L_) = 5.5 cm.

At higher pH, the dye will be in the anionic form and in a stronger position to donate electrons to the Pt electrode and reduced reductant while it is in its (dye) excited state and ground state, respectively, leading to an enhancement in the cell current. The dyes are also solubilized strongly at very high pH values on the order of 12 to 14, leading to enhancement of the cell output.

The anionic nature of the dye at higher pH coupled with anionic SLS may retard the aggregation behaviour of the dye in solution; this results in higher diffusion and current because PG cells are diffusion controlled. It is reported that the photoreduction of dyes with different reductants at different pH values depends on the reduction potential of the days and the oxidation potential of the reductants. The redox potentials of dyes become more negative with increasing pH, as shown by the cyclic-voltammetric data, because dye reduction will be difficult at higher pH due to the less favorable protonation of the dye because the protonation states of the leuco dyes and dyes change over the pH range;^50^ also, preferential reduction of hydrogen cations at the cathode will lead to a decrease in cell power.

It is also reported that a minimum pH of 12 to 14 is necessary for dyes to be reduced and solubilized completely, supporting the use of pH 13 in the present study.^55^

Thus, the effects of pH on the PG cell can be explained by these facts. The performance of the cell is poor in acidic medium. This may be due to proton attachment to heteroatoms and double bonds in the dye and reductant, leading to poor electron donating power of the dye and reductant to the Pt electrode. In alkaline medium, this effect is absent, and anion formation of the dye and reductant enhances their electron donation power. At very high pH, OH^−^ (from the NaOH used in this system) may combine with the cationic reductant (formed upon electron donation from the reductant to the dye), inhibiting regeneration of the reductant in its original form and leading to poor performance of the cell.

In this study, the pH has not been stabilized. The electrical parameters have been reported against the initial pH of the mixture of solutions of dye, reductant, and NaOH. The cell has the highest performance at an optimum pH for a particular combination of dye and reductant; therefore, study at a broader pH interval is not necessary.

### 3.6. Effects of the size of the Pt electrode on cell performance

The size of the Pt electrode affects the cell performance. Therefore, optimization of the size of the Pt electrode for optimal cell performance is of special significance. The effects of variation of the Pt electrode area on the cell were studied by constructing four photogalvanic cells while maintaining all factors in common except the Pt electrode area. It is a previously reported fact that a Pt electrode area of 0.08 cm^2^ (0.4 cm × 0.2 cm) is more effective for cell performance.^19^ Therefore, the size variation of Pt was studied near this value. Under the observed effects of the electrode area, the *i*_max_, *i*_sc_, *P*_pp_, and CE values were found to be highest for the Pt electrode area of 0.08 cm^2^ (0.4 cm × 0.2 cm). For electrodes with areas larger than 0.08 cm^2^, the cell parameters were found to decrease with increasing electrode area (Table 5, Sec. 14 of the ESI†). For the observed effect of the electrode area, better cell parameters were found for small electrodes; this is due to their relatively lower hindrance to diffusion of ions because photogalvanic cells are based on an ion diffusion mechanism.

**Table 5:** Effects of the size of the Pt electrode on cell performance^a^

Electrical parameters of the PG cell	Size of the Pt electrode			
	0.06 cm^2^ (0.2 × 0.3)	0.08 cm^2^(0.4 × 0.2)	0.16 cm^2^(0.4 × 0.4)	0.32 cm^2^(0.4 × 0.8)
*i*_max_ (μA)	5200	6000	5000	3500
*i*_sc_ (μA)	3000	3600	3100	2500
Potential at power point (mV)	570	685	700	705
Photocurrent at power point (μA)	1100	1200	1150	700
*P*_pp_ (μW)	627	822	805	493.5
Cell charging time (min)	36	40	55	61
CE (%)	19.39	20.41	11.61	3.64
FF	0.193	0.206	0.24	0.246

a At [formic acid] = 1.4 × 10^−3^ M, [metanil yellow dye] = 1.1 × 10^−4^ M, [SLS] = 1.4 × 10^−4^ M, pH = 13.76, Pt electrode area = 0.4 cm × 0.2 cm, light intensity = 10.4 mW cm^−2^, diffusion length (*D*_L_) = 5.5 cm.

Pt was used because authenticated data exists on its efficacy in photogalvanic cells. It is inert and very resistant to corrosion. It is also readily available in a ready-to-use form and has robust ability to withstand damage. The size of Pt used is very small, so it also provides economic competitiveness. However, in future, various aspects of the use of other low-cost alternatives, such as carbon nanostructures, metal sulfides, metal alloys, and conducting polymers may be studied for solar power generation through photogalvanic cells.

### 3.7. Effects of the diffusion length (*D*_L_) on cell performance

The distance between SCE and the Pt electrode (defined as the diffusion length) also affects the cell performance. Therefore, the study of the variation of the diffusion length for optimal cell performance is of special significance. The effects of variation of the diffusion length on PG cells were studied by constructing four photogalvanic cells with all factors in common except the diffusion length (separation between the centres of the two arms of the H-cell). The diffusion length significantly affects the performance of photogalvanic cells because they are based on the diffusion of ionic species. It has been observed that with an increase in the diffusion length, the photocurrent shows an increase and the potential shows a decreasing trend (Table 6, Sec. 15 of the ESI†). As the diffusion length increases, the current increases, probably because the conductivity of the dye increases due to the increase in the volume of solution between the electrodes. The potential decreases with diffusion length. This may be because the concentration gradient disturbs the dye layer (double layer) on the Pt electrode. When the diffusion length is small, the concentration gradient factor is decreased and the potential is increased.

**Table 6:** Effects of the diffusion length (*D*_L_) on cell performance^a^

Electrical parameters of the PG cell	Diffusion length (*D*_L_)			
	4.5 cm	5.0 cm	5.5 cm	6.0 cm
*i*_max_ (μA)	5450	5600	6000	6100
*i*_sc_ (μA)	2950	3250	3600	3700
Potential at power point (mV)	715	700	685	600
Photocurrent at power point (μA)	900	1100	1200	1250
*P*_pp_ (μW)	643.5	770	822	750
Cell charging time (min)	69	55	40	40
CE (%)	19.33	19.7	20.41	18.47
FF	0.25	0.213	0.206	0.205

a At [formic acid] = 1.4 × 10^−3^ M, [metanil yellow dye] = 1.1 × 10^−4^ M, [SLS] = 1.4 × 10^−4^ M, pH = 13.76, Pt electrode area = 0.4 cm × 0.2 cm, light intensity = 10.4 mW cm^−2^.

### 3.8. Effects of the distance between the illuminating source and the Pt electrode (*i.e.*, illuminating intensity) on cell performance

The distance from the illuminating source to the Pt electrode also affects the cell performance because the intensity of the illumination of the electrolyte varies with distance. In other words, it can be said that the distance between the illuminating source and the Pt electrode affects the intensity of the photons striking the photosensitive materials present in the electrolyte. Once the cell is charged by irradiation with a certain light intensity, it retains power even in the dark. The re-irradiation of this cell (which already has stored power) with a different light intensity will not provide correct information about the electrical output corresponding to the different light intensity. Therefore, a single cell is not sufficient for correct study of the effects of light intensity on the cell. Therefore, the effects of variation of the distance between the illuminating source and Pt electrode have been studied indirectly by using illuminating sources with different wattages [*i.e.*, incandescent lamps of 500 W, 200 W, 100 W, and 60 W; the corresponding intensities (mW cm^−2^) are 26, 10.4, 5.2, and 3.1, respectively]. This is because the ultimate aim of solar cells is their use in natural sunlight, where the distance between the sun and the Earth is fixed. In daily life, we cannot significantly change the distance between a solar cell and the sun. The only factor that matters is the variation of the intensity of the sun, which varies geographically and with time at a certain place on Earth. Therefore, the distance was kept constant (10 cm) and the lamps were changed to perform the experiment. There is a previously reported study on the effects of variation of light intensity on a cell by constructing various photogalvanic cells with all factors in common except the light intensity. In the same pattern, the study of the effects of variation of the light intensity on the cell by constructing four photogalvanic cells with all factors in common except the light intensity has been performed in the present work. These four cells are identical with respect to diffusion length, Pt electrode area, and concentrations of dye, reductant, surfactant and NaOH. The optimized cell performance was obtained at the illuminating intensity of 10.4 mW cm^−2^ (*i.e.*, the 200 wattage bulb). The photocurrent and photopotential showed increasing behaviours with increasing light intensity (Table 7, Sec. 16 of the ESI†). Increased light intensity increases the number of photons per unit area (incident power) striking the dye (photo sensitizer) molecules around the platinum electrode; therefore, there is an increase in the electrical output. At lower light intensity, the number of photons may be few in comparison to the dye molecules, leading to fewer numbers of dye molecules for electron donation to the Pt electrode. As the light intensity increases, the number of dye molecules for electron donation to the Pt electrode increases; hence, the electrical parameters also increase. At a very high light intensity, the performance of the cell decreases, probably for these reasons: (i) the dye molecules are limited in number, so a large number of photons remain unutilized, (ii) there is a relatively lower increase in power but a high increase in intensity, which leads to lower efficiency because the intensity is in the denominator of the formula of conversion efficiency, and (iii) higher intensity causes higher heating effects on the cell, leading to relatively poor performance of the cell.

**Table 7:** Effects of the distance between the illuminating source and the Pt electrode (*i.e.*, illuminating intensity) on cell performance^a^

Electrical parameters of the PG cell	Illuminating intensity (mW cm^−2^)			
	3.1	5.2	10.4	26
*i*_max_ (μA)	1800	4000	6000	7500
*i*_sc_ (μA)	1200	2600	3600	4200
Potential at power point (mV)	500	627	685	696
Photocurrent at power point (μA)	350	550	1200	1500
*P*_pp_ (μW)	175	344.8	822	1044
Cell charging time (min)	106	60	40	25
CE (%)	14.11	18.31	20.41	11.54
FF	0.20	0.221	0.206	0.23

a At [formic acid] = 1.4 × 10^−3^ M, [metanil yellow dye] = 1.1 × 10^−4^ M, [SLS] = 1.4 × 10^−4^ M, pH = 13.76, Pt electrode area = 0.4 cm × 0.2 cm, *D*_L_ = 5.5 cm.

### 3.9. Retrieval of stored power from the PG cell in the dark (performance of the cell in the dark)

The retrieval of stored power from the PG cell was studied to evaluate the inherent power storage capacity of the cell. The performance of each cell was studied in the dark in the absence of illumination at a characteristic power (maximum power extractable from the cell) at a characteristic external load resistance (2066.66 Ω). With time, the values of retrieved power, potential and current decrease in the dark (Table 8, Fig. S2†). The cell continuously supplies power until its complete discharge. The power decreases to half (93 μW) of its initial value (186 μW) in the half-time (105 minutes). Also, in the present case, the inherent power storage capacity of the PG cell was observed to be 105 minutes. Even after 105 minutes, the cell continued to supply power in the dark as a result of the retrieval of stored power. It is also very clear from Fig. S2† that the magnitude of power retrieved from the PG cell in the dark does not decrease abruptly but very slowly, and it gradually maintains a nearly constant value for many minutes. Here, the meaning of inherent storage is that the photogalvanic cell does not require any external power storage device but has the capacity of storing power in the form of species (semi-reduced dye, leuco-reduced dye) present in the liquid electrolyte. There is no deposition of charge on any electrodes to play a role in the power storage capacity of these cells. This is verified by the fact that the illuminated electrolyte lacking electrodes starts giving current as soon as the electrodes are dipped in the illuminated solution. The charging of the electrolyte with a set of electrodes and removal of this set of electrodes from solution followed by insertion of an entirely fresh set of electrodes also gives current. In this way, the charges responsible for the power storage may reside in the electrolyte.

**Table 8:** Retrieval of stored power from the PG cell in the dark at a characteristic external load resistance of 2066.66 Ω (performance of the PG cell in the dark)

Time (min)	Current (μA)	Potential (mV)	Power (μW)
0	300	620	186
10	300	615	184.5
20	300	600	180
30	250	508	127
40	250	500	125
50	250	480	120
60	200	600	120
70	200	570	114
80	200	550	110
90	200	530	106
100	200	505	101
105	200	465	93 (*t*_0.5_ 105 min)
110	200	490	98

It is highly unbelievable that a PG cell will be able to supply power after 105 min (half-time) in the dark due to residual triplet-excited states because its (triplet state) life is too short to exist for such a long time. More probably, the retrieval of power for a long time can be attributed to some species which are relatively more stable in the ground state; in the present case, this may be the accumulated two-electron-reduced dye (*i.e.*, the leuco-form of the dye). The accumulated leuco-form of the dye can exist for a long time and will probably contribute to the current generation until it is extinguished. The relatively more stable triplet excited state plays an indirect role in the power storage because its longer life (in comparison to the life of the singlet excited state) provides a higher probability of its interaction with the reductant and, in turn, a higher probability of formation of the leuco dye form.

The existence of leuco dye in the ground state can be explained on the basis of the fact that organic molecules have only two types of excited states (singlet and triplet) which can show intersystem crossing, radiative deactivation (luminescence), radiationless deactivation, and photochemical reactions. In the present case, the leuco form of the dye is formed by quenching as a result of reduction upon electron acceptance from the reductant. This means that the excited singlet/triplet states of the dye no longer remain excited once they have obtained electrons from the reductant.

The cell functions as a reversible device (can undergo cycles of charging and discharging) with a natural element of imminent irreversibility as per the 2^nd^ law of thermodynamics, which states that ideally, no process is reversible because the forces of resistance, dissipation of energy, friction, *etc.* are imminent in all processes. As far as the present device of a photogalvanic cell is concerned, the discharged cell can be re-charged with the original fabrication components (components which were integral to the initial charging of the cell). Factors such as photodecay of the dye sensitizers, mineralization of the dye, the sacrificial nature of the reductant, corrosion of electrodes, and evaporation of the electrolyte prevent the re-generation of a cell power output equal to the cell power output generated immediately preceding the charging process. However, the preliminary data in the present study show that the cell can be charged a second time to produce almost the same level of power (*P*_pp_ 820 μW) as was produced in the initial charging (*P*_pp_ 822 μW). It is expected that the charging of the cell in successive cycles will not repeat the same level of power generation, as occurs in all types of batteries, such as Li ion batteries and lead storage batteries. The beauty of photogalvanic cells is that the photodecay products of the dye will also provide harvesting of sunlight because the illuminating source encompasses the whole UV-visible spectral range and is thus capable of photo-exciting species present in the decayed electrolyte. However, an extensive study on the cyclability of the photogalvanic behavior of this cell will require a year of time with the view of long term application in daily life; this was neither planned in the present study, nor is it possible now to perform this study for want of timely submission of the revised manuscript to this journal. We thank the honourable reviewers for pointing out this aspect of the study, and the same will be undertaken at an extensive level in future research work.

There will also be an effect of air on the solar energy conversion efficiency of photogalvanic cells. The performance of the cell will be adversely affected by the presence of air in the cell solution due to the presence of oxygen because oxygen molecules can quench the triplet state of the sensitizer through the mainly diffusion-controlled process. The oxygen is excited through the spin-forbidden slow process of intersystem crossing (ISC) involving the conversion of the ground triplet state to the excited singlet state (^1^Δ_g_ singlet state 0.9773 eV above the triplet ground state and ^1^Σ^+^ singlet 1.6268 eV above the triplet ground state). The life of the excited singlet state of the dye molecules is too short (on the order of 10^−8^ seconds) to collide with oxygen to cause its own quenching (quenching of singlet dye). On the other hand, the triplet state of the dye has a relatively long life (on the order of 10^−3^ seconds) and can undergo collision with oxygen molecules, leading to its own quenching (quenching of triplet dye). This is because the emission rate from the triplet dye (phosphorescence) is spin forbidden and hence has rate constants in the range of 10 to 10^3^ dm^3^ mol^−1^ s^−1^, whereas oxygen quenching may take place at faster rate constants on the order of 10^9^ dm^3^ mol^−1^ s^−1^.^56^ The Stern–Volmer equation model states that dynamic quenching mainly occurs by the quencher diffusing through the solution and interacting with the dye, resulting in deactivation of the excited state.^57^ The higher the atmospheric pressure of the air over the cell solution, the higher the amount of oxygen in the cell solution, leading to increased deterioration of the efficiency of the cell. The temperature increase and low viscosity of the solvent will favour diffusion, leading to more quenching of the triplet dye sensitizer.

Therefore, to decrease the quenching of the triplet dye by oxygen, the technique of de-aerating the cell solution with nitrogen gas can be used. The use of a more viscous solvent and lower temperature for decreasing dye quenching is estimated to be counter-productive; this is because it will decrease the diffusion of species in solution and in turn decrease the cell performance because these cells are diffusion-controlled devices. Another technique is the use of a dye with an energetic triplet state that is different from and does not overlap with the energy of excitation of the oxygen molecule; this may also be helpful to address the problem of dye quenching. Coincidentally, this was observed in the present study of the use of metanil yellow as a sensitizer in a photogalvanic cell. Metanil yellow has a singlet excited state energy of ∼2.99 eV (equivalent to a 414 nm band gap between the HOMO and LUMO) and a triplet excited state energy of ∼2.78 eV (the triplet state energy is generally 5% to 10% lower than the singlet state energy for dye molecules). On the other hand, the ^1^Δ_g_ singlet state of oxygen is 0.9773 eV above its triplet ground state, and the ^1^Σ^+^ singlet state of oxygen is 1.6268 eV above its triplet ground state. From this fact, it is clear that the energy of the triplet state of MY dye and the excitation energy of oxygen do not overlap, leading to prevention of dye quenching by oxygen. This is supported by a previous report, which stated that metanil yellow does not produce detectable amounts of singlet oxygen. One additional benefit of preventing dye quenching is that it will favour reversibility of the cell function.^58^ The causes of the decrease of dye quenching have been suggested to be steric factors and interaction processes involving non-bonding electrons that are competitive with pi-system charge-transfer complexation.^59^ It should be noted that this aspect of the study of the effects of air on cell performance was neither planned nor performed in the present study. However, the authors are tempted to plan such a study in the future; meanwhile, we suggest this study to other researchers.

### 3.10. Discussion

PG cells have been extensively studied under low-intensity artificial sunlight. In the present work, the optimum cell performance in terms of electrical parameters is *i*_max_ 6000 μA, *i*_sc_ 3600 μA, *V*_oc_ 1105 mV, *P*_pp_ 822 μW, and CE 20.41%. The results in the present work are higher with respect to previous work^16,27–29,37^ and are also consistent with the aim of undertaking this work to further increase the electrical performance of PG cells. The results of the present study are sufficiently novel to report advancement over previous results (Table 9), *viz.*, (a) *P*_pp_ 649.6 μW, CE 8.12%, and power storage capacity 59 minutes,^37^ (b) *P*_pp_ 158.9 μW, *V*_oc_ 960.0 mV, *i*_sc_ 350.0 μA, and CE 1.52%,^27^ (c) *P*_pp_ 93.15 μW, *V*_oc_ 919 mV, *i*_sc_ 210 μA, and CE 0.8967%.^60^ The reasons for the superior results in the present work may be the use of metanil yellow dye photosensitizer, formic acid reductant, higher pH (*i.e.*, 13.76) and a small Pt electrode (0.4 cm × 0.2 cm). The lower mass of metanil yellow (MW 376.39) coupled with the higher pH of the alkaline medium (*i.e.*, 13.76) facilitates diffusion and transfer of electrons to the Pt electrode and, in turn, the greater current and power observed in the present work. The anionic SLS surfactant enhances the solubility and electron ejection capacity of the dye photo-sensitizer. The lower Pt electrode creates less hindrance to the diffusion of molecules in solution in the cell. The higher pH not only facilitates the solubility and reduction of dye sensitizers but also enhances their preferred reduction at the counter electrode by diminishing the reduction of hydrogen ion (H^+^) at the counter electrode. The formic acid reductant has greater reducing capacity than fructose in the electrolytic conditions of the PG cell, as indicated by their respective reduction potentials (*vs.* SHE) of +0.3725 V and +0.3925 V. The work reported by Saini *et al.*^27^ uses a large Pt electrode area (1 cm × 1 cm), lower pH (*i.e.*, 12.75), and relatively heavy orange G dye sensitizer (MW 452.38). The work reported by Koli^37^ uses a relatively lower pH (*i.e.*, 13.6), fructose reductant, and relatively heavy fast green FCF dye sensitizer (MW 808.86). Thus, the observed cell performance based on the metanil yellow dye-formic acid-SLS is encouraging enough for the ultimate aim of the development of applicable and affordable PG cells in the future.

**Table 9:** Comparison of the results of the present manuscript with previously published literature data on photogalvanic systems (cells)^a^

Sensitizer-reductant-surfactant photogalvanic system	Open-circuit potential (*V*_oc_)	Short-circuit current (*i*_sc_)	Power at power point (*P*_pp_)	Conversion efficiency (CE)
Fast green FCF sensitizer-fructose reductant^b^^28^	1066 mV	380 μA	138.6 μW	1.33%
Lissamine green B sensitizer-ascorbic acid reductant-SLS surfactant^16^	850 mV	375 μA	106.08 μW	1.02%
Safranine O sensitizer-EDTA reductant-SLS surfactant^29^	1052 mV	1700 μA	364.7 μW	8.93%
Fast green FCF sensitizer-fructose reductant^c^^37^	1048 mV	2250 μA	649.6 μW	8.12%
Orange G sensitizer-EDTA reductant-SLS surfactant^27^	960.0 mV	350.0 μA	158.9 μW	1.52%
Biebrich scarlet sensitizer-ascorbic acid reductant-Tween 60 surfactant^60^	919 mV	210 μA	93.15 μW	0.8967%
Metanil yellow sensitizer-formic acid reductant-SLS surfactant^d^	1110 mV	6000 μA	822 μW	20.41%

a SLS (sodium lauryl sulfate); EDTA (ethylenediaminetetraacetate sodium salt).

b 10.4 mW cm^−2^ emitted from a 200 watt incandescent tungsten bulb.

c 100 mW cm^−2^ (average value) natural sunlight as available in daily life.

d Present work.

On further comparing the performance of the PG cell in the present work with respect to previously published literature, it was noted that an earlier published study on the SLS surfactant in a PG cell at artificial and low-intensity light emitted from a 200 watt incandescent bulb reported *i*_sc_ 380 μA, *P*_pp_ 138.6 μW and CE 1.33%.^28^ A study on the photogalvanic effect in a lissamine green B sensitizer-ascorbic acid reductant-SLS system reported *i*_sc_ 375 μA, *V*_oc_ 850 mV, and CE 1.02%.^16^ A study on the photogalvanic effect in an EDTA-safranine O-SLS system reported *i*_sc_ 1700 μA, *P*_pp_ 364.7 μW and CE 8.93%.^29^ The present manuscript carries this work ahead and reports greater advancement over earlier reported work by employing the metanil yellow photo-sensitizer-formic acid couple in the presence of a cell performance enhancer surfactant at relatively high pH under artificial and low illumination intensity.

Photogalvanic solar cells are advantageous compared to other devices, such as conventional silicon solar cells, dye sensitized solar cells,^61^ perovskite solar cells,^62^ fuel cells, lithium batteries,^63^ supercapacitors^64^ and photocatalytic water splitting^65^ in the following ways: (i) photogalvanic solar cells have inherent power storage capacity with simultaneous solar energy conversion. They can be charged in sunlight, and without any further technical addition, this stored power can be retrieved directly from the cell. (ii) The fabrication of these cells is very facile and also inexpensive. (iii) These cells can be charged even in diffused and very low illumination sources.

## 4. Conclusion

The present study of metanil yellow dye as a photosensitizer and formic acid as a reductant in the presence of sodium lauryl sulphate surfactant and sodium hydroxide alkaline medium shows greatly enhanced electrical performance (over earlier results for similar cells) of *P*_pp_ 822 μW, *i*_sc_ 6000 μA, *V*_oc_ 1110 mV, CE 20.41%, and *t*_0.5_ 105 minutes. On the basis of the redox potential, as observed in the present study and published data, the photo-generation of the current in the metanil yellow-formic acid photogalvanic system follows the same mechanistic route reported for other sensitizer-reductant couples used in PG cells. From these facts, it may be concluded that the use of the metanil yellow dye sensitizer-formic acid reductant-SLS chemical combination is a promising alternative for the fabrication of highly efficient PG cells for harvesting solar power with simultaneous power storage in an eco-friendly fashion. The eco-friendly nature of the metanil yellow dye sensitizer-based PG cells can be enhanced by the removal of exhausted cell solution containing this dye by a decolourization process using an electrochemical reduction method.^41^

## Conflicts of interest

There are no conflicts to declare.

## Supplementary Material

RA-009-C8RA10014D-s001
