# Supplementary material for: Simultaneous electrochemical solar power generation and storage using metanil yellow-formic acid as a new sensitizer-reductant couple in photogalvanic cells
Source: RSC Adv. 2019 Mar 6;9(13):7560–74. doi: 10.1039/c8ra10014d (PMC9061176; doi:10.1039/c8ra10014d)
Supplement: RA-009-C8RA10014D-s001 [file RA-009-C8RA10014D-s001.pdf]

Electronic Supplementary Material (ESI) for RSC Advances.
This journal is © The Royal Society of Chemistry 2019

SUPPLEMENTARY INFORMATION FOR

Simultaneous electrochemical solar power generation and storage by using the Metanil Yellow-Formic acid as a new sensitizer-reductant couple in the photogalvanic cells

Pooran Koli, Yashodhara Dayma and Ramesh Kumar Pareek, Department of Chemistry, Jai Narain Vyas University, Jodhpur -342001, Rajasthan, India*

1. DESCRIPTION OF THE COMPOUNDS USED

(A) METANIL YELLOW: The chemical compound Metanil Yellow (70% Assay-purity) has been used as photo-sensitizer. Characteristics of the Metanil Yellow dye are M.F. $C_{18}H_{14}N_3NaO_3S$, M.W. 375.78 g/mol, λ_{max} 414 nm and C.I. No. 13065. It is an anionic azo dye. Its solubility is 25g/l at 20 ^{0}C.

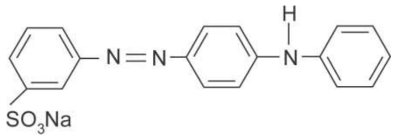

Chemical structure of the Metanil Yellow dye

(B) FORMIC ACID: It has been used as reductant in this research work for solar energy conversion and storage. Chemical formula: HCOOH; Molar mass: 46.03gmol^{-1}; IUPAC Name: Methanoic acid; Appearance: colourless fuming liquid; Density: 1.220 g/ml; Melting point: 8.0 ^{0}C; Boling point: -100.8 ^{0}C; Dipole moment: 1.41 D (gas); Molecular shape: Planar.

(C) SODIUM LAURLY SULPHATE (SLS): It has been used as micellar agent in this research work for solar energy conversion and storage.SLS is an anionic surfactant. It lowers the surface tension of the aqueous solutions. Formula**:** C12H25SO4.Na; Molecular weight: 288 g/mol; Appearance: White or cream-colored solid; odour: Odourless; Density: 1.01gcm^{-3}; Melting point: 206^{0}C.

* *Corresponding author, +91 291 2614162; poorankoli@rediffmail.com*;**poorankoli@yahoo.com**

(D) SODIUM HYDROXIDE (NaOH): It has been used as alkaline medium in this research work for solar energy conversion and storage.Formula: NaOH; Molar mass: 39.99 g/mol; Appearance: White, opaque crystals; odour: odourless; Density: 2.13g/cm^3; Melting point: 318 ^{0}C; Solubility: soluble in water.

(E) OXALIC ACID: It has been used as a standard reagent for standardizing the NaOH solution in this research work for solar energy conversion and storage. Formula: $C_2H_2O_4$; Molar mass: 90.03g/mol; Odour: Odourless; Melting point: 189 to 191^{0}C; Solubility: soluble in ethanol and diethyl ether; colour: White solid.

(F) PHENOLPHTHALEIN: It has been used as an indicator in standardisation of the alkali in this research work for solar energy conversion and storage. Formula: $C_{20}H_{14}O_4$; Molar mass: 318.33 gmol^{-1}; Appearance: White power; Density: 1.277g/cm^3; Solubility: Soluble in ethanol and ether; and slightly soluble in DMSO. It belongs to a class of the dyes called phthalein dyes.

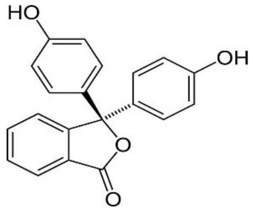

Chemical structure of the phenolphthalein

2. *DESCRIPTION OF THE ELECTODES USED*

(A) COMBINATION ELECTRODE: Combination electrode means a single cylinder containing both the reference electrode, and a glass membrane electrode.

(B) PLATINUM ELECTRODE: It is a metal electrode made of the Platinum (Pt) metal. It is a most inert electrode suitable for the efficient electron exchange between electrode electrolyte solutions. Under strong alkaline conditions, they actually function as oxidation electrodes. It provides a surface for oxidation and reduction reaction.

3. *PREPARATION OF THE SOLUTIONS:* All these solutions have been prepared in single distilled water and kept in amber colour vessels to protect them from sunlight.

4. EXPERIMENTAL METHOD: The experimental set up is as below (**Figure S1**) -

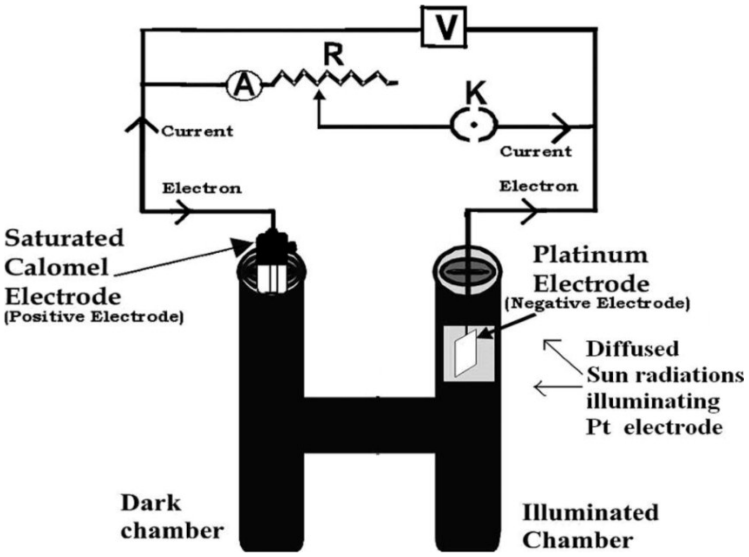

Figure S1. *Photogalvanic cell set up. A- Micro-ammeter, K-Key, R-Resistance, V- Digital pH meter.*

The photogalvanic cell is made of externally blackened H- shaped glass tube filled with a solutions of Photosensitizer (M/500), reductant (Formic acid M/100), surfactant (Sodium Lauryl Sulphate M/10) and Sodium hydroxide (1M) (**Figure S1**) to keep the total volume of reaction mixture always 68 ml. The cell will be H-shaped with two arms. Both arms shell be blackened from outside keeping a window in one arm (this arm called illuminated chamber with dipped Pt electrode). A platinum electrode is dipped into one window arm, and combination electrode is dipped in other arm. The terminals of the electrode are connected to a digital pH meter and the micro-ammeter [19-20]. The system is then exposed to the artificial sunlight (emitted from the incandescent lamp, i.e., tungsten lamp of different wattage). The study of the current voltage characteristics and determination of the power point was accomplished by making use of the resistance variation device called carbon (log 470 K).

The fill factor (FF) was calculated as $(V_{pp}\times i_{pp})/(V_{oc} \times i_{sc})$, and conversion efficiency (CE) was calculated as $(V_{pp} \times i_{pp} \times FF \times 100\ \%)/(A \times I)$; where, ‘A’ is Pt electrode area, the ‘I’ is illuminating intensity in $mWcm^{-2}$. $V_{pp} \times i_{pp}$ is power at power point (P_{pp}) expressed in mW.

The size of open window (illuminated area of the cell) of illuminated arm has not been used for calculating the efficiency as- (i) the light diffraction, scattering, and edge effects effectively illuminate the larger solution area, (ii) the PG cell is diffusion controlled, therefore, only those dye molecules which are nearer to Pt anodic electrode shall be able to reach the electrode within their excited lifetime. It means that the number of such dye

molecules shall be determinable with respect to Pt area but not the window size; (iii) the natural sunlight is available naturally and free of cost. So to illuminate the small or large area of the cell does not affect the cost and environment. But, the use of small or large Pt electrode affects the cost and environment. Therefore, the use of small Pt electrode area will be less costly and more eco-friendly as low demand for Pt will need less production of Pt leading to reduction in pollution; (iv) The use of Pt anode area for calculation of the efficiency is also supported by the published work of scientists Murthy *et al.* [**34**], who have used cross-sectional area of the anode for the calculation of conversion efficiency of photogalvanic cell and have given a formula to calculate it, i.e., CF = (V_{pp} x i_{pp} x FF x 100 %)/(PA); and (v) the electrical performance is found independent of the area of illuminating window.

Here, it is to be kindly noted that the conversion efficiency and fill factors are two of the main parameters for judging the performance of the solar cells. Naturally, the higher efficiency should also accompany with the higher FF. But in case of the photogalvanic cells, the FF is relatively low due to certain reasons like photo decay of dye, etc. Because of this, the efficiency and FF does not seem coordinated but divergent to each other. For, the photogalvanic cells, the efficiency is high but the FF low. So to correlate them and to make efficiency linked to FF, the present formula as described in the manuscript has been used, and which is also substantiated by already published literature [**19**].

5. MEASUREMENT OF THE PHOTOPOTENTIAL: A digital pH meter (Systronics Model-335) is used to measure the potential at different time intervals. The whole system was first placed in the dark till a stable potential is obtained. It is a dark potential. Then, the Pt electrode was illuminated with sunlight and the reaction mixture was allowed to reach equilibrium potential, again which was also measured. The difference between the dark potential and the equilibrium potential is termed as photo-potential (mV). The potential of the circuit in short-circuit conditions (i.e., current zero in circuit) is called open-circuit potential (V_{oc}).

6. MEASUREMENT OF THE PHOTOCURRENT: Micro-ammeter was used to measure the current in the circuit.

7. MEASUREMENT OF THE pH: The pH of the reaction mixture was measured by a digital pH (Systronics Modal: 335) and was also calculated by the used amount of sodium hydroxide solution by the following formula as follows- pH = 14- log [OH^-].

8. CURRENT VOLTAGE (i-V) CHARACTERISTICS OF THE CELL: The current-voltage (i-V) characteristics of the cell were studied using an external load (log 470 K) in the circuit. The highest current obtained at the zero resistance of the circuit after the full charging of the cell is termed as maximum current (i_{max}), which after some time decreases to an equilibrium vale called short-circuit current (i_{sc}). In other words, the i_{sc} is defined as the current of the circuit at zero potential. The potential at the short-circuit current is noted, then the resistance of the circuit is gradually decreased to note the potential value at each of the decreasing current value, and finally the potential is noted at zero current. The product of the current and its corresponding potential gives the power value of the cell. The highest product value of the power is called the power at power point of the cell (or the maximum power extractable at corresponding circuit resistance that is characteristics external load). The current and potential at power at power point are termed as current at power point and potential at power point, respectively. The graph between the current and its corresponding potential is termed as i-V curve of the cell. The curve is generally in 'U' shape. The maxima of the curve shows the power at power point and the current and potential values corresponding to the maxima are termed as the current at power point and potential at power point, respectively.

9. PERFORMANCE AND CONVERSION EFFICIENCY OF THE CELL:

The performance of the cell was determined at its power point from the rate of fall in the power of the cell after removing the source of light. An external load applied to have the current and the potential values equivalent to the value at the power point. Then, the time taken in fall of the power of the cell to its half value is recorded. It is denoted as half time ($t_{1/2}$) which has been taken as a measure for the performance of the cell in dark (storage capacity of the cell). The higher the $t_{1/2}$ means higher the power storage capacity of the cell.

10. THE EFFECT OF THE VARIATION OF THE METANIL YELLOW DYE CONCENTRATION: The effect of the variation of the metanil yellow dye concentration on metanil yellow-formic acid- SLS photogalvanic system has been studied by fabricating the fourphotogalvanic cells in H-shaped glass tube vessel. The concentrations [of the reductant formic acid, surfactant SLS, alkali NaOH (i.e. pH)], diffusion length, Pt electrode size and combination electrode are same for all four cells, but the dye concentrations are different in different cells. For fabricating a cell, the 40 ml of 1 M NaOH, 10 ml of M/10 SLS, and 10 ml of M/100 Formic acid has been taken in each cell consisting of H-shaped glass tube with the Pt electrode area = 0.4 cm × 0.2 cm, Light intensity = 10.4 $mWcm^{-2}$, and Diffusion length (D_L) = 5.5 cm. Along with this amount of the solutions of NaOH, SLS, and Formic acid; the

3.6 ml of M/500 metanil yellow dye solution + 4.4 ml single distilled water was taken in the 1st cell; the 3.8 ml of M/500 metanil yellow dye solution +4.2 ml single distilled water was taken in the 2nd cell; the 4.0 ml of M/500 metanil yellow dye solution +4.0 ml single distilled water was taken in 3rd cell; and the 4.2 ml of M/500 metanil yellow dye solution +3.8 ml single distilled water was taken in 4th cell. In each cell, the total volume of solution including the water was 68 ml. The resultant concentrations was as, [Dye] = 0.9×10^{-4} M, [Formic acid] = 1.4×10^{-3} M, pH = 13.76, and [SLS] = 1.4×10^{-2} M for the 1st cell; [dye] = 1.0×10^{-4} M, [Formic acid] = 1.4×10^{-3} M, pH = 13.76, and [SLS] = 1.4×10^{-2} M for the 2nd cell; [dye] = 1.1×10^{-4} M, [Formic acid] = 1.4×10^{-3} M, pH = 13.76, and [SLS] = 1.4×10^{-2} M for the 3rd cell; and [dye] = 1.2×10^{-4} M, [Formic acid] = 1.4×10^{-3} M, pH = 13.76, and [SLS] = 1.4×10^{-2} M for the 4th cell. The cell shows the highest electrical output for [Dye] = 1.1×10^{-4} M.

11. THE EFFECT OF THE VARIATION OF THE FORMIC ACID REDUCTANT CONCENTRATION: The effect of the variation of the formic acid reductant concentration on the metanil yellow-formic acid- SLS photogalvanic system has been studied by fabricating the four photogalvanic cells. The concentrations [of the metanil yellow dye sensitizer, SLS surfactant, NaOH alkali (i.e. pH)], diffusion length, Pt electrode size and combination electrode are same for all four cells, but the formic acid concentration are different in different cells. For fabricating a cell, the 40 ml of 1M NaOH, 4.0 ml of M/500 dye, and 10 ml of M/10 SLS has been taken in each cell consisting of H-shaped glass tube with the Pt electrode area = 0.4 cm × 0.2 cm, Light intensity = 10.4 $mWcm^{-2}$, and Diffusion length (D_L) = 5.5 cm. Along with this amount of solutions of NaOH alkali, Dye sensitizer, and SLS surfactant; the 8.0 ml of M/100 formic acid solution + 6.0 ml single distilled water was taken in the 1st cell; the 10 ml of M/100 formic acid solution + 4.0 ml single distilled water was taken in the 2nd cell; the 12 ml of M/100 formic acid solution +2.0 ml single distilled water was taken in the 3rd cell; and the 14 ml of M/100 formic acid solution + 0.0 ml single distilled water was taken in the 4th cell. In each cell, the total volume of solution including the single distilled water was 68 ml. The resultant concentrations was as, [Dye] = 1.1×10^{-4} M, [Formic acid] = 1.1×10^{-3} M, pH = 13.76, and [SLS] = 1.4×10^{-2} M for the 1st cell; [dye] = 1.1×10^{-4} M, [Formic acid] = 1.4×10^{-3} M, pH = 13.76, and [SLS] = 1.4×10^{-2} M for the 2nd cell; [dye] = 1.1×10^{-4} M, [Formic acid] = 1.7×10^{-3} M, pH = 13.76, and [SLS] = 1.4×10^{-2} M for the 3rd cell; and [dye] = 1.1×10^{-4} M, [Formic acid] = 2.0×10^{-3} M, pH = 13.76, and [SLS] = 1.4×10^{-2} M for the 4th cell.

12. THE EFFECT OF THE VARIATION OF THE SLS SURFACTANT CONCENTRATION: The effect of the variation of the SLS surfactant concentration on the

metanil yellow-formic acid-SLS system has been studied by fabricating the four photogalvanic cells. The concentrations [of dye, formic acid, NaOH (i.e. pH)], diffusion length, Pt electrode size and combination electrode are same for all four cells, but the surfactant concentrations are different in different cells. For fabricating a cell, the 40 ml of 1M NaOH, 4.0 ml of M/500 Dye, and 10 ml of M/100 formic acid has been taken in each cell consisting of the H-shaped glass tube with the Pt electrode area = 0.4 cm × 0.2 cm, Light intensity = 10.4 $mWcm^{-2}$, and Diffusion length (D_L) = 5.5 cm. Along with this amount of solutions of NaOH, dye, and formic acid; the 6.0 ml of M/10 SLS solution + 8.0 ml single distilled water was taken in the 1st cell; the 8.0 ml of M/10 SLS solution + 6.0 ml single distilled water was taken in the 2nd cell; the 10 ml of M/10 SLS solution + 4.0 ml single distilled water was taken in the 3rd cell; and the 12 ml of M/10 SLS solution + 2.0 ml single distilled water was taken in the 4th cell. In each cell, the total volume of the solution including the single distilled water was 68 ml. The resultant concentrations was as, [Dye] = 1.1×10^{-4} M, [Formic acid] = 1.4×10^{-3} M, pH = 13.76, and [SLS] = 0.8×10^{-2} M for the 1st cell; [dye] = 1.1×10^{-4} M, [Formic acid] = 1.4×10^{-3} M, pH = 13.76, and [SLS] = 1.1×10^{-2} M for the 2nd cell; [dye] = 1.1×10^{-4} M, [Formic acid] = 1.4×10^{-3} M, pH = 13.76, and [SLS] = 1.4×10^{-2} M for the 3rd cell; and [dye] = 1.1×10^{-4} M, [Formic acid] = 1.4×10^{-3} M, pH = 13.76, and [SLS] = 1.7×10^{-2} M for the 4th cell.

13. THE EFFECT OF THE VARIATION OF THE NAOH (i.e. pH) CONCENTRATION:

The effect of the variation of the NaOH (i.e. pH) concentration on the metanil yellow-formic acid- SLS photogalvanic system has been studied by fabricating the four photogalvanic cells. The concentrations [of the dye sensitizer, formic acid reductant, and SLS surfactant], diffusion length, Pt electrode size and combination electrode are same for all cells, but the NaOH (i.e. pH) concentrations are different in the different cells. For fabricating a cell, the 4.0 ml of M/500 ml Dye, 10 ml of M/10 SLS, and 10 ml of M/100 Formic acid has been taken in each cell consisting of H-shaped glass tube with Pt electrode area = 0.4 cm × 0.2 cm, Light intensity = 10.4 $mWcm^{-2}$, and Diffusion length (D_L) = 5.5 cm. Along with this amount of solutions of Dye, SLS, and Formic acid; the 38 ml of 1M NaOH solution + 6.0 ml single distilled water was taken in the 1st cell; the 40 ml of 1 M NaOH solution + 4.0 ml single distilled water was taken in the 2nd cell; the 42 ml of 1 M of NaOH solution + 2.0 ml single distilled water was taken in the 3rd cell; and the 44 ml of 1M NaOH solution + 0.0 ml single distilled water was taken in the 4thcell. In each cell, the total volume of the solution including the single distilled water was 68 ml. The resultant concentrations was as, [Dye] = 1.1×10^{-4}

M, [Formic acid] = 1.4 × 10^{-3} M, pH = 13.74, and [SLS] = 1.4 × 10^{-2} M for the 1st cell; [dye] = 1.1 × 10^{-4} M, [Formic acid] = 1.4×10^{-3} M, pH = 13.76, and [SLS] = 1.4×10^{-2} M for the 2nd cell; [dye] = 1.1 × 10^{-4} M, [Formic acid] = 1.4×10^{-3} M, pH = 13.78, and [SLS] = 1.4 × 10^{-2} M for the 3rd cell; and [dye] = 1.1 × 10^{-4} M, [Formic acid] = 1.4 × 10^{-3} M, pH = 13.80, and [SLS] = 1.4×10^{-2} M for the 4th cell.

14. THE EFFECT OF VARIATION OF PT ELECTRODE AREA ON THE CELL PERFORMANCE: The effect of variation of Pt electrode area on the cell performance has been studied by constructing four photogalvanic cells having all factors common except Pt electrode area. The concentrations and values of other cell fabrication parameters were as explained earlier in para 10 -13.

15. THE EFFECT OF VARIATION OF THE DIFFUSION LENGTH ON THE CELL PERFORMANCE: The effect of variation of the diffusion length on the cell performance has been studied by constructing four photogalvanic cells having all factors common except diffusion length (separation between centres of two arms of H-cell). The concentrations and values of other cell fabrication parameters were as explained earlier in para 10-13.

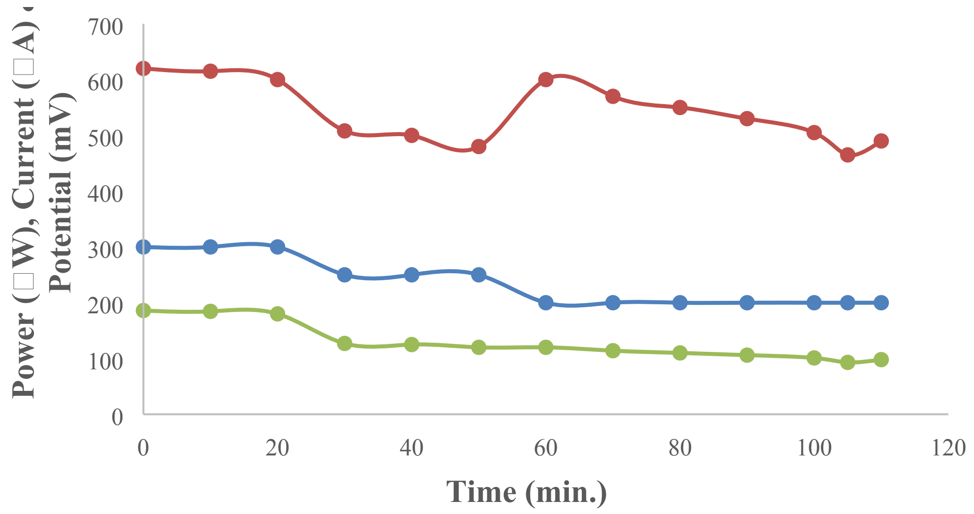

Figure S2. *Study of cell performance in the dark (the retrieval of the stored power from the cell), (1) potential vs time (uppermost curve), (2) current vs time (middle curve), (3) power vs time (lowest curve).*

16. THE EFFECT OF VARIATION OF DISTANCE BETWEEN ILLUMINATING SOURCE AND PT ELECTRODE: The effect of variation of distance between illuminating source and Pt electrode has been studied indirectly by using illuminating source of different wattage [i.e., incandescent lamps of 500 W, 200 W, 100 W, and 60 W; corresponding intensities ($mWcm^{-2}$) respectively as 26, 10.4, 5.2, and 3.1]. The concentrations and values of other cell fabrication parameters were as explained earlier in para 10 -13.

Table S1. The values of the potential and power at various current values obtained by varying the circuit resistance (i-V characteristics of the cell).

Current (μA)	Potential (mV)	Power (μW)
3600	22	86.4
3400	40	136
3200	50	160
3000	110	330
2800	118	330.4
2600	133	345.8
2400	210	504
2200	265	583
2000	350	700
1800	405	729
1600	485	776
1400	565	791
1200 (**i_{pp}**)	685 (**V_{pp}**)	822 ***(P_{pp}), CE = 20.41 %, FF = 0.206***
1000	716	716
800	770	616
600	850	510
400	980	392
200	1050	210
0	1099	0

**The results are so obtained for metanil yellow- formic acid- SLS system based PG cell are V_{oc} = 1105 mV, i_{sc}=3600 μA, i_{pp}=1200μA, V_{pp}=685 mV, P_{pp}=822 μW, CE=20.41%, FF=0.206.*
